# Mass spectrometry-based N-glycosylation analysis in kidney disease

**DOI:** 10.3389/fmolb.2022.976298

**Published:** 2022-08-17

**Authors:** Weifu Ren, Qi Bian, Yan Cai

**Affiliations:** ^1^ Shanghai Institute of Precision Medicine, Ninth People’s Hospital, Shanghai Jiao Tong University School of Medicine, Shanghai, China; ^2^ Department of Nephrology, Changhai Hospital, Naval Medical University, Shanghai, China

**Keywords:** mass spectrometry, N-glycosylation, kidney disease, biomarker, LC-MS, MALDI-MS

## Abstract

Kidney disease is a global health concern with an enormous expense. It is estimated that more than 10% of the population worldwide is affected by kidney disease and millions of patients would progress to death prematurely and unnecessarily. Although creatinine detection and renal biopsy are well-established tools for kidney disease diagnosis, they are limited by several inevitable defects. Therefore, diagnostic tools need to be upgraded, especially for the early stage of the disease and possible progression. As one of the most common post-translational modifications of proteins, N-glycosylation plays a vital role in renal structure and function. Deepening research on N-glycosylation in kidney disease provides new insights into the pathophysiology and paves the way for clinical application. In this study, we reviewed recent N-glycosylation studies on several kidney diseases. We also summarized the development of mass spectrometric methods in the field of N-glycoproteomics and N-glycomics.

## 1 Introduction

Kidney disease is increasingly recognized as a global health concern and brings an overwhelming public burden. At present, patients with kidney disease amount to more than 850 million people worldwide, and the annual medical care expense is as high as 35 billion dollars which is still insufficient (Rosenberg, 2020). Considering that the 5-year survival rate of patients receiving dialysis is even lower than most malignancies (Rosenberg and Ibrahim, 2019), kidney disease calls for early discovery, diagnosis, and treatment to avoid the occurrence of end-stage renal disease (ESRD). Nowadays, serum creatinine, as the most representative marker, is subject to its nonspecific, delayed properties and lacks sensitivity to tubular injury (Zhang and Parikh, 2019). Kidney biopsy cannot be routinely and repeatedly applied in clinical work due to a bleeding incidence of 70% (Hogan et al., 2016), even though it is the well-acknowledged gold standard in the diagnosis of kidney disease. Therefore, it is necessary to deepen the understanding of kidney disease and innovate diagnostic tools and treatment methods to achieve better prognosis.

**FIGURE 1:**
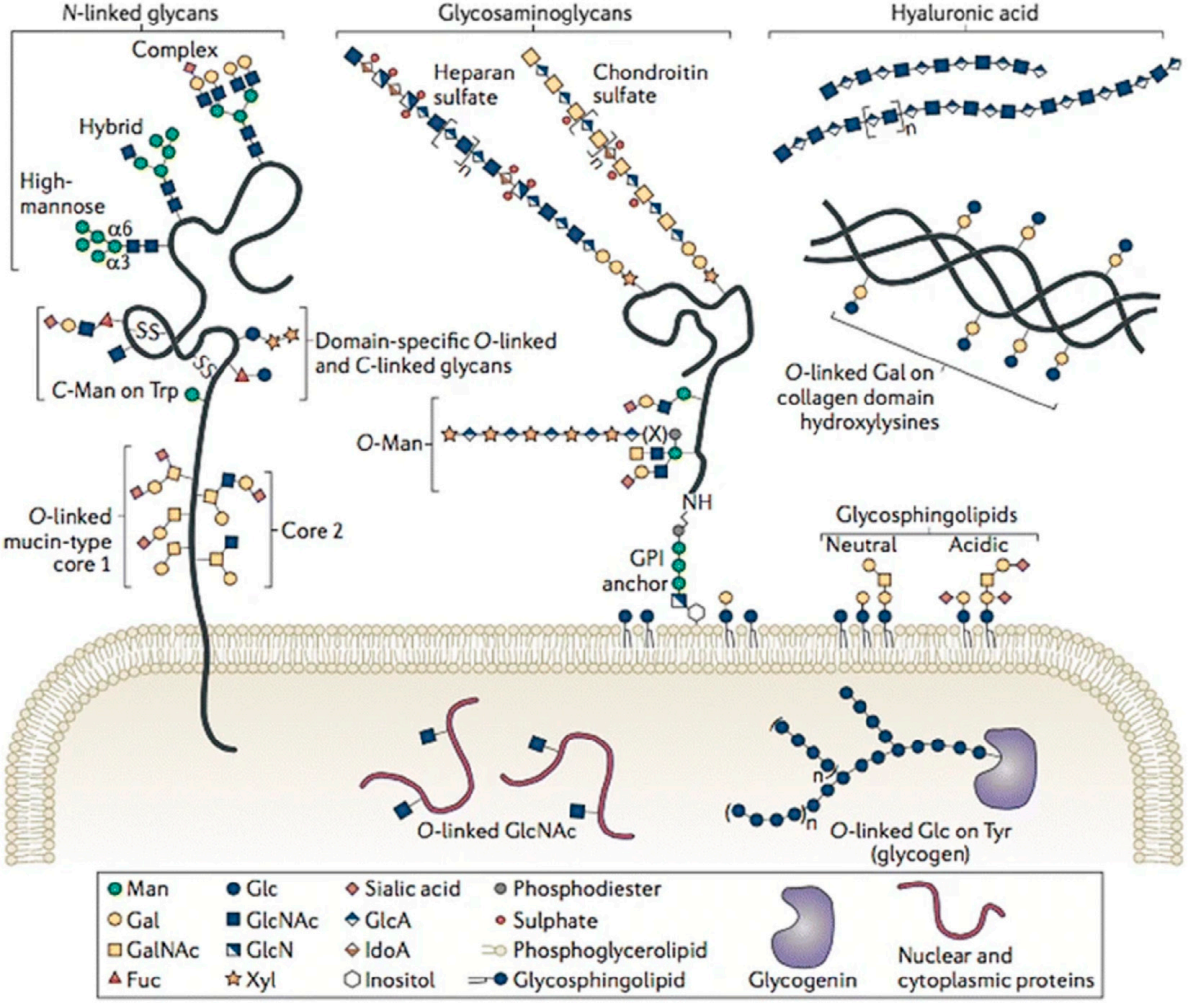
Variety of glycoconjugates in human cells. Among them, N- and O-glycans are the most frequent post-translational modifications of proteins, which are attached to certain Asn or Ser/Thr residues, respectively. Reproduced from Corfield et al., (2017). Eukaryotic protein glycosylation: a primer for histochemists and cell biologists. Histochemistry and Cell Biology, 147(2), 119–147. DOI: 10.1007/s00418-016-1526-4 under the terms of the Creative Commons Attribution 4.0 International License (http://creativecommons.org/licenses/by/4.0/).

Glycosylation is one of the most frequent and structurally diverse post-translational modifications of proteins in eukaryotes, which can be classified into two main categories: N-glycosylation and O-glycosylation (Schjoldager et al., 2020). As the most commonly studied glycosylation type, N-glycosylation refers to the covalent attachment of a glycan moiety to the nitrogen atom of asparagine (Asn) residues by a β-1N linkage in the motif Asn-X-serine/threonine (Ser/Thr), where “X” is any amino acid except proline. In humans, N-glycans are classified into three basic types: high-mannose, hybrid, and complex types, all of which begin with N-acetylglucosamine (GlcNAc) β1-Asn and contain a GlcNAc2 mannose3 core (Stanley et al., 2022) (Figure 1). Protein N-glycosylation plays crucial roles in various biological processes, such as protein folding, cell signaling, host–pathogen interaction, and immune response. Aberrant protein N-glycosylation is closely associated with many diseases, including inflammation, rheumatoid arthritis, cancer, and even the ongoing COVID-19 pandemic (Mereiter et al., 2019; Steffen et al., 2020; Watanabe et al., 2020).

Glycoproteins cannot function well until proper glycosylation. O-Glycosylation and N-glycosylation both contribute to maintaining normal renal function and influence the pathology of the kidney. O-Mannosyl glycans regulate glomerular filtration by mediating the interface between podocyte foot processes and the glomerular basement membrane (GBM) (Reily et al., 2019). Abnormal galactose-deficient O-glycopeptides which induce autoimmune response participate in the process of immune-mediated kidney disease, such as IgA nephropathy (IgAN) (Pattrapornpisut et al., 2021). Similarly, proper N-glycosylation is indispensable for the right membrane localization of several essential component proteins, such as nephrin, podocin, and Crumbs2; enables their interactions with other molecules; and further sustains the glomerular filtration barrier (GFB) function (Yan et al., 2002; Serrano-Perez et al., 2018; Möller-Kerutt et al., 2021). Abnormal N-glycosylation participates in the pathogenesis of kidney disease by various mechanisms, for instance, influencing protein maturity, exposing protein immunogenicity, and promoting inflammatory response (Yu et al., 2017; Besse et al., 2019; Haddad et al., 2021). Moreover, N-glycosylation holds immense clinical practice value not only in the field of diagnosis and prognosis with samples of plasma/serum, urine, urinary exosomes, dialysate, and tissue (Staubach et al., 2016; Noro et al., 2017; Ferrantelli et al., 2018; Mise et al., 2018; Sinha et al., 2020; Alves et al., 2021) but could also be used as direct and effective therapeutic targets (Wang et al., 2017; Li et al., 2018; Zhong et al., 2020).

**FIGURE 2:**
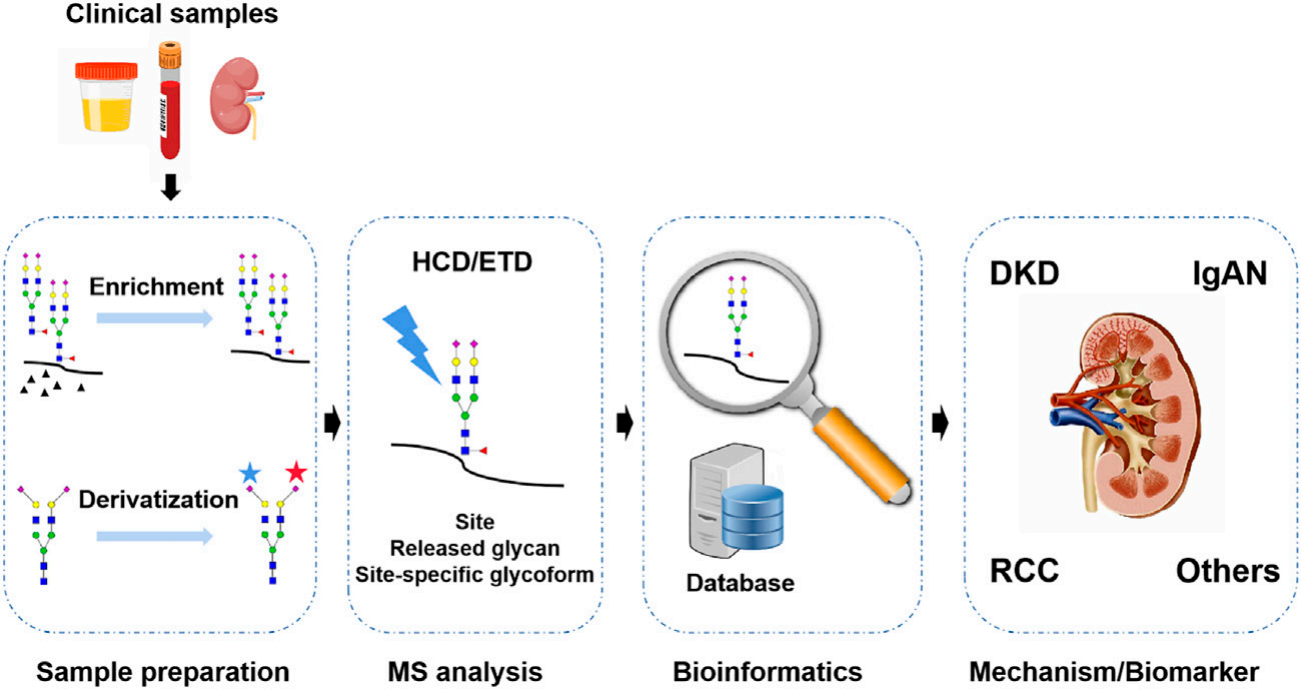
MS-based N-glycosylation analysis in kidney disease is the focus of this review.

For N-glycosylation analysis in kidney disease, lectin array and/or HPLC with optical detection were two commonly used techniques, but they lacked the sufficient ability of glycan structure characterization and only provided limited N-glycoproteome/N-glycome coverage and analytical throughput in biomarker discovery and mechanism study (Bermingham et al., 2018; Kawakita et al., 2021). At present, mass spectrometry (MS) has proven to be a powerful tool for the global analysis of N-glycosylation in complex biological samples. Compared with other technologies, the superiority of MS lies in its high sensitivity, rapidity, and ability to provide structural information on glycans, which could meet the requirement of clinical N-glycosylation profiling in a large cohort study (Dang et al., 2019). MS-based N-glycoproteomics and N-glycomics are gaining great interest in clinical research including in kidney disease (Peng et al., 2018; Pan et al., 2020). In this review, recent progresses in MS-based approaches for N-glycoproteomics and N-glycomics are briefly discussed, including N-glycopeptide/N-glycan preparation, MS analysis, and bioinformatics platforms and their application in kidney disease (Figure 2).

## 2 MS-based N-glycoproteomics and N-glycomics technologies

### 2.1 General workflow of MS-based clinical N-glycosylation analysis

**FIGURE 3:**
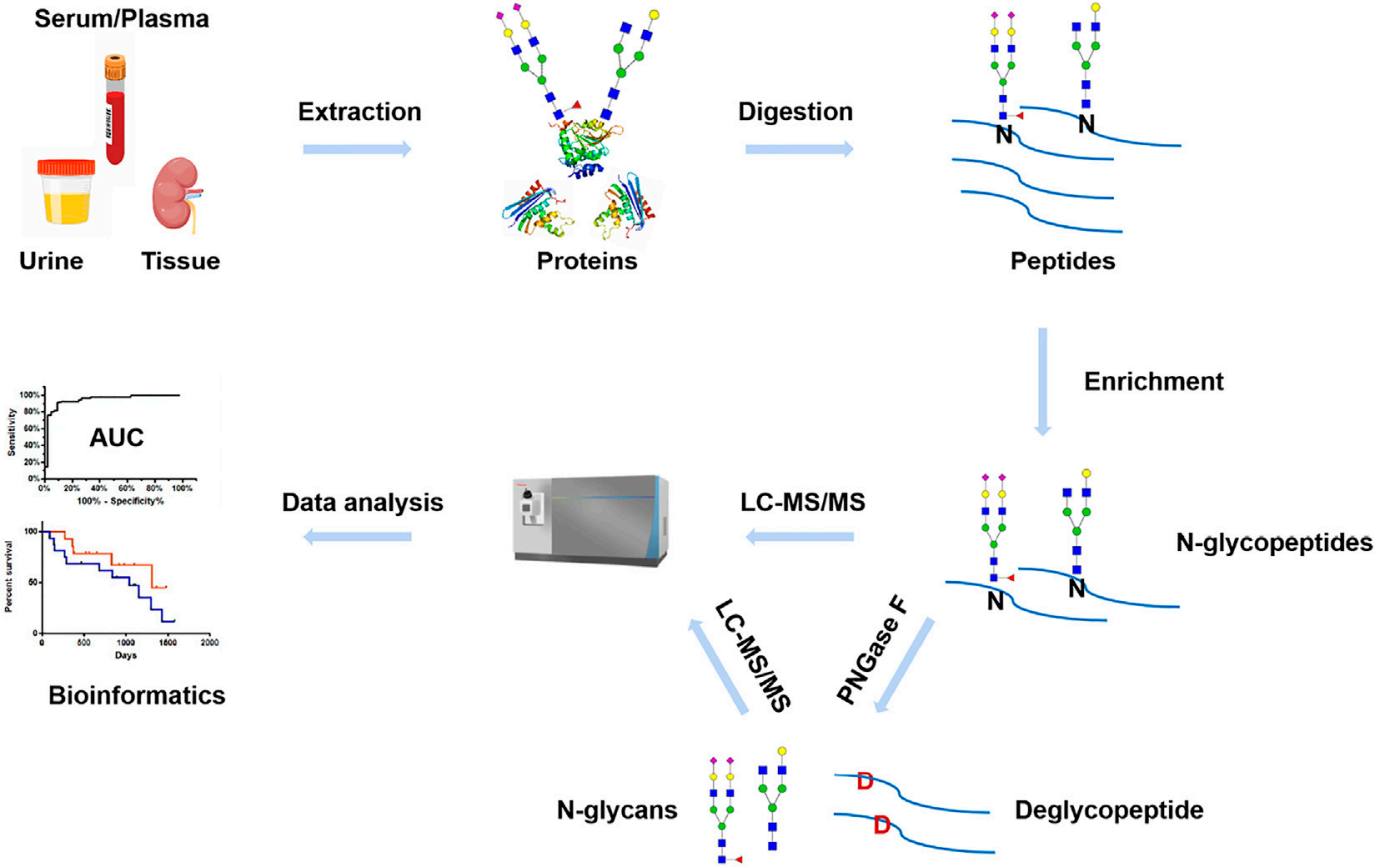
Workflow of MS-based N-glycosylation analysis in clinical samples.

MS has been widely used for N-glycosylation analysis in various clinical samples, such as serum/plasma, urine, and biopsy tissue. Typically, a general workflow of MS-based clinical N-glycosylation analysis includes the following steps (Figure 3): proteins are extracted from the clinical samples by different methods, such as precipitation with organic solvent, ultracentrifugation, and affinity columns. After protein denaturation and tryptic digestion, N-glycopeptides are selectively enriched from the complex digests. For N-glycosylation site mapping, after removing N-glycans by PNGase F, deglycosylated peptides are subjected to LC-MS analysis. In parallel, the released N-glycans are characterized by MS under native status or after chemical derivatization. For site-specific N-glycosylation analysis, intact N-glycopeptides undergo different dissociation methods during LC-MS analysis and are interpreted by bioinformatics tools. The comprehensive analysis of protein N-glycosylation in clinical samples will help the investigation of disease development and provide an alternative yet critical tool in early diagnosis and treatment.

### 2.2 Sample preparation

Typically, the types of biological samples for clinical testing mainly include whole blood, serum/plasma, urine, biopsy tissue, and cerebrospinal fluid. As for kidney disease, serum/plasma, urine, and biopsy tissue are commonly collected. Prior to MS, owing to the low abundance and poor ionization efficiency, the extraction of N-glycopeptide/N-glycan from the aforementioned complex clinical samples is crucial (Riley et al., 2021). In this section, we will give a brief overview of two key steps in N-glycopeptide/N-glycan sample preparation, including N-glycopeptide/N-glycan enrichment and derivatization.

#### 2.2.1 N-glycopeptide/N-glycan enrichment

Analysis of N-glycopeptides by MS is usually hampered by their low abundance, poor ionization efficiency, and suppression by numerous non-glycopeptides. Therefore, for precise identification and quantitation of N-glycoproteome in clinical samples, specific enrichment of N-glycopeptides prior to MS analysis is an essential step to increase their MS detection sensitivity (Palaniappan and Bertozzi, 2016). So far, there are three commonly used strategies in N-glycopeptide enrichment: 1) enrichment based on lectin and affinity enrichment. Recently, three different types of lectins, namely, concanavalin A, wheat germ agglutinin, and agglutinin RCA120, were applied to enrich urinary N-glycoproteome of clear cell renal cell carcinoma (ccRCC) patients at different stages (Santorelli et al., 2020). Although a lectin-based enrichment approach presents good specificity, the main limitation is that the versatility of each lectin type is narrow and the combination of multiple lectins with different affinities to certain glycan subsets has to be used for in-depth N-glycoproteome coverage (Santorelli et al., 2020; Yu et al., 2020). 2) The second is hydrophilic interaction chromatography (HILIC). Depending on the hydrophilic interactions between glycans and the matrix, HILIC enrichment is widely used to effectively and unbiasedly enrich N-glycopeptides (Qing et al., 2020). Fang et al. (2020) developed a streamlined pipeline for multiplexed quantitative site-specific N-glycoproteomics, including cell lysis, protein aggregation capture PAC-based clean-up and proteolysis, TMT-labeling, and zwitterionic HILIC enrichment. This optimized workflow allows the fast preparation of TMT-labeled N-glycopeptides and enables 1-day sample preparation, showing the potential application in large cohort clinical samples. 3) The third strategy is chemical covalent interactions. Boronic acid-based enrichment is a versatile method by which N-glycopeptide could be selectively but reversibly enriched. However, the interactions between glycans and boronic acid are relatively weak, causing a loss of low-abundance glycopeptides. Recently, efforts have been made to enhance glycan–boronic acid interactions by the synthesis of multimeric boronic acids or dendrimeric boronic acid-functionalized materials (Xiao et al., 2018a; Guy et al., 2019). Hydrazide chemistry and its derivatives, such as oxime click chemistry and thiazolidine chemistry, were another covalent interaction-based method for N-glycopeptide enrichment. By using hydrazide Affi-gel, Liljedahl et al. (2016) enriched N-glycopeptides from the kidney tissue of diabetic mouse models, revealing that N-glycoprotein differences could be a clue to dissimilarities in type 1 diabetes mellitus (T1DM) and type 2 diabetes mellitus (T2DM) at later stages of diabetic kidney disease (DKD). The main advantage of these approaches is their high selectivity to N-glycopeptides, but they were limited in N-glycosylation site mapping because of the loss of N-glycan information (Zhang et al., 2003; Zhang et al., 2014; Cai et al., 2020).

Generally, upon releasing N-glycans from the glycoprotein/glycopeptide by PNGase F, N-glycome is enriched from the mixture through size exclusion, HILIC, porous graphitized carbon, or tagging-assisted strategies (Lu et al., 2016). To explore the potential of N-glycan in early monitoring of the progression of T2DM toward chronic kidney disease (CKD), Adua et al. (2018) purified plasma N-glycans by HILIC-based solid phase extraction on a commercial AcroPrep GHP filter plate. From a clinical viewpoint, a simple, fast, and cost-friendly N-glycan preparation workflow is in need. Peng et al. (2019a) developed a streamlined strategy for the rapid and selective analysis of serum N-glycome, where sterilized cotton was used as a matrix for glycoprotein absorption, rapid deglycosylation, and highly selective N-glycan enrichment. Compared to traditional methods, sample preparation could be shortened from 24 to 2.5 h, and the selectivity toward glycan was improved ∼100 fold than commercially available purification microtips.

Overall, due to the structural diversity of glycoform and the heterogeneity of N-glycosylation sites, there is no universal N-glycopeptide/N-glycan enrichment strategy. For clinical researchers, it is better to be aware of the strengths and limitations of each method and make an appropriate choice for their own N-glycosylation study (Riley et al., 2021).

#### 2.2.2 Linkage-specific derivatization

Chemical derivatization is a powerful approach to improving MS detection of N-glycopeptide/N-glycan (Lu et al., 2016). To further investigate the detailed structure of glycan, for example, linkage isomers of sialylated N-glycans (a-2,3- and a-2,6-linked sialic acids) that play a vital role in various physiological and pathological processes including kidney disease (Babal et al., 1996), linkage-specific derivatization is emerging as a new tool but still remains a challenge. In this part, we focused on the recent linkage-specific analysis of sialylated N-glycans/N-glycopeptides.

Based on the differences in reactivity, sialylated N-glycan isomers were added to isomeric-specific chemical groups by various derivatization methods, such as amidation and esterification. The mass shift introduced by derivatization between two isomeric glycans allows the distinction of these glycan isomers in MS analysis (Peng et al., 2021a). The representative strategy was a two-step derivatization method developed by Wuhrer et al. In the first step, α-2,6-linked sialic acid formed a stable dimethylamide through the reaction with dimethylamine, whereas α-2,3-linked sialic acid formed an unstable lactone. In the second step, α-2,6-linked sialic acid was left unreacted, whereas the lactone on α-2,3-linked sialic acid converted to a stable amide (Holst et al., 2016; Nishikaze et al., 2017; Hanamatsu et al., 2019). In this strategy, however, the difference in chemical composition after derivatization will lead to a difference in ionization efficiency and the chromatographic behavior between glycan isomers.

To overcome this circumstance, Peng et al. (2019b) developed a stable-isotope-based sequential selective derivatization approach for the differentiation and relative quantification of isomeric-linked sialic acids containing N-glycans. The use of isotopic methylamine hydrochloride reagents minimized the bias in ionization efficiency caused by the difference in chemical composition and chromatographic behavior. By optimizing the derivatization conditions, stable-isotope-based sequential derivatization was also successfully applied to the analysis of sialyl-linkage isomers on intact N-glycopeptide levels (Peng et al., 2021b). In addition, without the assistance of derivatization, linkage-specific intact N-glycopeptides can be unambiguously identified and relatively quantified using their characteristic fragment ions in ion mobility mass spectrometry (IM-MS) (Feng et al., 2021).

### 2.3 Mass spectrometric method

Due to the complex microheterogeneity of the glycoprotein, the comprehensive analysis of protein N-glycosylation mainly includes N-glycosylation site profiling, and N-glycan composition and linkage analysis at each site. Currently, there are two strategies for MS-based N-glycoproteomics: N-glycosylation site profiling and intact N-glycopeptide analysis.

#### 2.3.1 N-glycosylation site profiling

For N-glycosylation site profiling, N-glycopeptide is treated with peptide-N-glycosidase (PNGase F) to remove glycans. Deamidation occurs at the N-glycosylation site, which converts Asn residues into aspartic acids and serves as a mass tag (+0.98 Da or +2.99 Da in H_2_^18^O) for N-glycosylation site identification by MS (Liang et al., 2022). The use of endo-β-N-acetylglucosaminidases (endo-D, F, or H) is an alternative method to assist N-glycosylation site profiling, by which the glycosidic bond between the two GlcNAc is cleaved, leaving a single GlcNAc core attached to its peptide backbone as a + 203.0794 Da tag for site identification (Hägglund et al., 2007; Lowenthal et al., 2016). For N-glycosylation site profiling in some glycoproteins, multiple proteases are required for efficient digestion to increase sequence coverage. For instance, a combination of trypsin and Glu-C was used to improve the identified number of N-glycosylation sites of purified clusterin that is heavily glycosylated and upregulated in ccRCC tumors (Gbormittah et al., 2015).

#### 2.3.2 Released N-glycan

After PNGase F treatment, in parallel to N-glycosylation site profiling, the released N-glycans are analyzed by MS under native status or after chemical derivatization, providing complementary N-glycome information.

Compared to LC-ESI-MS, MALDI-MS features in high-throughput analysis of N-glycan in clinical samples and better salts compatibility (Harvey, 2021). Recently, using a combination of 96-well plate-based sample preparation and MALDI-MS detection, total plasma N-glycome patterns were successfully analyzed from two large cohorts (*n* = 611 in DiaGene and *n* = 394 in Hoorn Diabetes Care System) of nephropathy (Memarian et al., 2021). However, native N-glycan profiling by MALDI-MS usually faces low detection sensitivity due to poor ionization efficiency and sample loss caused by a laborious labeling process. To address these limitations, on-target glycan derivatization methods were developed to simplify sample preparation and enhance N-glycan signals by the incorporation of the hydrophobic group and/or a permanent charge. A single-step protocol on-MALDI-target N-glycan nonreductive amination with 2-aminobenzoic acid (2-AA) was described. 2-AA can function as both a labeling reagent and an excellent MALDI matrix, thereby simultaneously increasing ionization efficiency, eliminating tedious sample preparation, and avoiding sample loss, but this method is only applicable to limited amounts of glycoproteins (Hronowski et al., 2020).

Additionally, MALDI-MS imaging (MSI) is also commonly used to provide the spatial distribution of N-glycans across tissue sections (McDowell et al., 2021). In 2019, MSI was applied to analyze the N-glycans of multiple human kidney tissues and tissue microarrays representing normal, polycystic kidney disease and multiple ccRCC stages, which provided N-glycan compositional differences between tumor and nontumor regions (Drake et al., 2020). For formalin-fixed paraffin-embedded tissue sections, the detection sensitivity of native glycans is limited by serious interference with the matrix of the tissue. On-tissue/*in situ* derivatization of N-glycans with Girard’s Reagent P (positively charged hydrazine reagent) significantly improved signal-to-noise ratios and N-glycome coverage, facilitating spatial visualization of disease-relevant N-glycans (Zhang et al., 2020).

#### 2.3.3 Intact N-glycopeptide analysis

Intact N-glycopeptides, also known as site-specific N-glycans, refer to the peptide backbone sequence, N-glycosylation site, and the corresponding glycoform at this site. Compared with the separate analysis of N-glycosylation sites and N-glycans, intact N-glycopeptide characterization is imperative yet more technically challenging.

For intact N-glycopeptide characterization, higher-energy collisional dissociation (HCD) and electron transfer dissociation (ETD) are two commonly used tandem MS fragmentation methods (Olsen et al., 2007; Riley and Coon, 2018). In recent years, stepped collision energy HCD (sceHCD) has emerged as a suitable alternative, where lower HCD collision energies are often advantageous for N-glycan fragmentation, while higher HCD collision energies tend to provide better peptide backbone fragmentation (Liu et al., 2017; Zeng et al., 2021). More recently, a combination of electron-transfer/higher-energy collisional dissociation (EThcD) and the sceHCD MS method was developed for site-specific N-glycosylation mapping of plasma IgG in CKD patients. This proposed method generated more informative fragment ions and higher spectral quality, thereby nearly doubling the depth of intact N-glycopeptide identification (Zhang et al., 2021).

For intact N-glycopeptide characterization by ETD, one of the great challenges is low dissociation efficiency due to the low charge density of N-glycopeptide precursors (Riley and Coon, 2018). Recently, chemical derivatization was used to improve the ETD efficiency by increasing the ion charge of N-glycopeptides. For example, N,N-dimethylethylenediamine (DMEN), a tertiary amine, was first introduced to label N-glycopeptides (Yang et al., 2019). By using DMEN derivatization, Sun et al. (2022) conducted the most comprehensive site-specific and subclass-specific N-glycosylation profiling of human serum IgG and revealed glyco-signatures for liver disease diagnosis. In addition, activated-ion electron transfer dissociation (AI-ETD), which uses concurrent infrared photoactivation, was also reported to improve intact N-glycopeptide fragmentation efficiency and N-glycoproteome coverage (Riley et al., 2019).

The strengths and limitations of conventional HCD, sceHCD, ETD, and EThcD for intact N-glycopeptide analysis were summarized and systematically discussed. Compared to HCD, sceHCD generally generates higher quality MS/MS spectra, but the number of identifications was similar. Compared to EThcD, both HCD and sceHCD showed much better performance in terms of the number of identifications, indicating that ETD-based methods might not be the first choice for large-scale N-glycopeptide profiling even if they can generate higher quality MS/MS spectra (Riley et al., 2020).

### 2.4 Bioinformatics platforms

Data analysis is the final yet key step in MS-based N-glycoproteomics/N-glycomics. Site-specific N-glycopeptide interpretation is more difficult because it requires simultaneous identification of peptide bone, N-glycan moiety, and N-glycosylation sites. Over the last decade, compared to existing search engines such as commercial Byonic and in-house GPQuest, the development of diverse bioinformatics solutions offered substantial increases in speed, accuracy, or sensitivity for large-scale intact N-glycopeptide analysis. Among them, pGlyco 2.0 has contributed significantly, as it first performs comprehensive quality control that included a false discovery rate (FDR) at three levels of glycans, peptides, and glycopeptides (Liu et al., 2017). In another software, the MSFragger-Glyco search engine used open and mass offset searches that greatly improved confidence for the identification of labile N-glycans (Polasky et al., 2020). Moreover, different from the aforementioned glycan database-dependent strategies, database-independent search strategies, such as the recently developed StrucGP and Glyco-Decipher, showed advantage in the identification of N-glycopeptides with unexpected/rare glycans or even glycans linked with modifications (Shen et al., 2021; Fang et al., 2022).

With the rapid expansion of released N-glycan datasets, the development of bioinformatics tools for N-glycomics data analysis is in steady progress. Recently, a detailed overview of software for N-glycomics data analysis and structural/experimental databases and repositories was described (Rojas-Macias et al., 2019). GlycoWorkbench is one of the most widely used tools for the computer-assisted annotation of mass spectra of N-glycans (Ceroni et al., 2008). Similarly, GRITS Toolbox provided another freely available software for processing, annotating, and archiving N-glycomics MS data (Weatherly et al., 2019). The main limitation of these two software is the lack of an algorithm for in-depth structural elucidation. Recently published GlycoDeNovo (Hong et al., 2017), Glycoforest (Horlacher et al., 2017), and GlySeeker (Xiao et al., 2018b) developed algorithms for structural analysis. Despite this, manual validation is still an inevitable step in spectrum interpretation. The use of FDR for quality control in N-glycome is in its infancy.

To date, most kidney disease-related N-glycoproteomics/N-glycomics data interpretations are based on manual extraction of previously identified N-glycopeptides/N-glycans. The development of the aforementioned search software would shed light on precise and high throughput site-specific N-glycosylation data analysis for a cohort study of kidney disease. Additionally, although the datasets of N-glycosylation were increasing, the creation of N-glycosylation-specific databases or repositories such as N-GlycositeAtlas for nephrology is still far behind (Sun et al., 2019).

## 3 N-glycosylation and kidney disease

Proper N-glycosylation is the necessary prerequisite to the function of glycoproteins which constitute the kidney structure. In GFB, it helps maintain the integrity of the GFB and avoid nephrotic syndrome. For example, N-glycans in nephrin expressed in the slit diaphragm of podocyte as an important transmembrane glycoprotein are vital in the trafficking of nephrin to the plasma membrane (Yan et al., 2002; Niculovic et al., 2019). Likewise, as for podocin, a podocyte-specific protein, N-glycosylation is not only essential for its biosynthesis, folding, and quality control but also of great value in the interaction with other proteins (Serrano-Perez et al., 2018).

**FIGURE 4:**
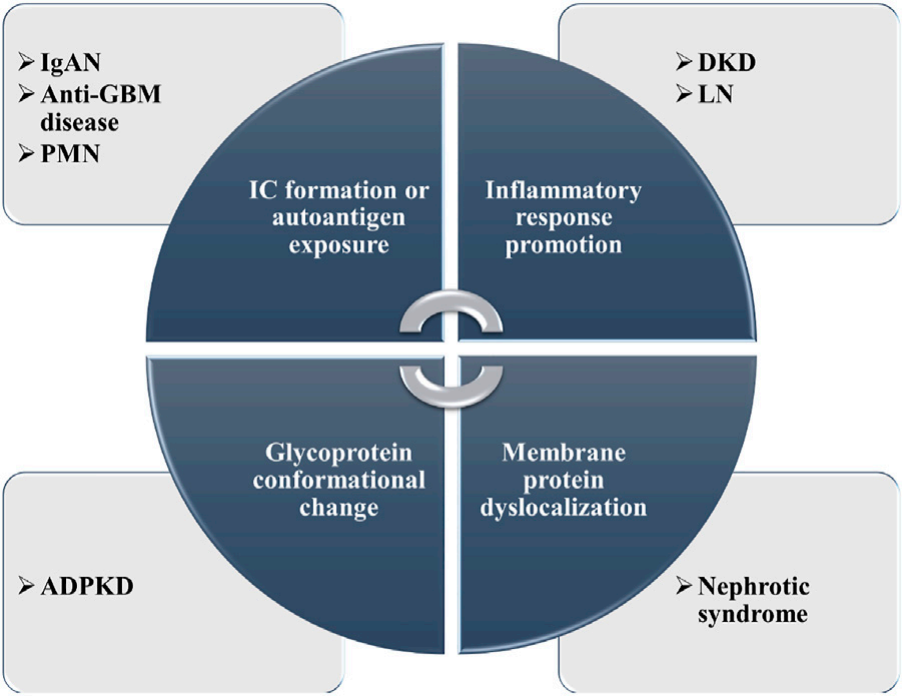
Summary of the possible pathogenesis of N-glycosylation in several kidney diseases.

With the rapid development of MS-based N-glycosylation analysis technologies and methods, large-scale datasets of N-glycosylation have provided new insights into pathophysiology investigation, early biomarker discovery, and effective treatment targets in different kidney diseases. In this section, the N-glycosylation study on several representative kidney diseases including DKD, IgAN, renal cell carcinoma (RCC), anti-GBM disease, autosomal dominant polycystic kidney disease (ADPKD), primary membranous nephropathy (PMN), and lupus nephritis (LN) will be discussed. Some of the research achievements are summarized in Table 1. The possible pathogenesis of N-glycosylation in several kidney diseases is also summarized (Figure 4).

**TABLE 1:** Summary of the representative MS-based N-glycosylation study in different kidney diseases.

Kidney disease type	Samples	Analytes	Analysis methods			Number	Findings	Reference
			Enrichment/derivatization	MS	Software			
DKD	Mouse kidney tissue	Deglycosylated N-glycopeptide	Hydrazide Affi-gel enrichment	LC-MS/MS	Progenesis QI	395 N-glycoproteins/3564 N-glycopeptides in the db/db mouse model and 505 N-glycoproteins/2602 N-glycopeptides in the STZ mouse model	N-glycoprotein differences (e.g., integrin-β1 and the sodium/glucose cotransporter-1) could be a clue to dissimilarities in T1DM and T2DM at later stages of DKD.	Liljedahl et al. (2016)
DKD, IgAN, MN	Human plasma IgG	N-Glycopeptide	NA	LC-MS/MS	Byonic	93, 73, 72, and 83 unique N-glycopeptides and 29, 27, 27, and 29 unique N-glycans in HC, MN, DKD, and IgAN, respectively, a total of 350 intact N-glycopeptides (106 IgG1, 111 IgG2, 67 IgG3, and 66 IgG4)	28 Aberrantly expressed IgG intact N-glycopeptides had the potential for the diagnosis of DKD, IgAN, and MN	Zhang et al. (2021)
DKD	Human urine	N-Glycopeptide	ConA agarose enrichment	LC-MS/MS	Mascot and scaffold	408 N-glycoproteins. 72, 107, and 123 differential proteins in the DKD stage of normoalbuminuria, microalbuminuria, and macroalbuminuria, respectively	Alpha-1-antitrypsin and ceruloplasmin are the two markers to distinguish microalbuminuria and normoalbuminuria. Pro-epidermal growth factor and prolactin-inducible protein were decreased in the macroalbuminuria stage	Guo et al. (2015)
DKD	Human plasma	N-Glycan	Ethyl esterification derivatization	MALDI-MS	Compass data analysis	73 and 68 N-glycans in two cohorts, respectively	15 N-glycan features were associated with DKD. Sialylation linkage type was associated with T2DM DKD.	Memarian et al. (2021)
IgAN	Human serum IgA	N-Glycopeptide	NA	LC-MS/MS	NA	39 N-glycopeptides. 36 N-glycans. 5 N-glycosylation sites: IgA1: N144, N340; IgA2: N131, N205, and N327	Multiple structural features of N-glycosylation of IgA1 and IgA2 were associated with IgAN and glomerular function	Dotz et al. (2021)
IgAN	Human serum IgA (a rare IgAN case)	N-Glycan	Permethylation	MALDI-MS	NA	22 N-glycans in the rare IgAN case, 18 N-glycans in the patients with circulating mIgA lacking renal involvement, and 20 N-glycans in the HC	mIgA deposition in the mesangial area is a rare disease. The patient had an abnormal monoclonal IgA N-glycan profile	Kaneko et al. (2010)
AC-associated IgAN	Human serum IgA1	N-Glycans	Permethylation	MALDI-MS	NA	19 N-glycans in the compensated cirrhosis, 19 N-glycans in advanced cirrhosis, 19 N-glycans in the primary IgAN, and 17 N-glycans in the HC	Both IgAN and AC feature abnormally glycosylated IgA1 and soluble CD89-IgA and IgG-IgA complexes. Common environmental factors may influence development of IgAN in susceptible individuals	Tissandié et al. (2011)
ccRCC	Human urine	Deglycosylated N-glycopeptide	Lectin-based enrichment	LC-MS/MS	Mascot	484, 513, and 479 N-glycopeptides in early stage ccRCC, advanced stage ccRCC, and non-ccRCC, respectively	CD97, COCH, and P3IP1 were up-expressed while APOB, FINC, CERU, CFAH, HPT, and PLTP were down-expressed in ccRCC patients	Santorelli et al. (2020)
ccRCC	Human serum clusterin	Deglycosylated N-glycopeptide and intact N-glycopeptide	NA	LC-MS/MS	SEQUEST	26 Site-specific N-glycopeptides. 7 N-glycosylation sites: N86, N103, N145, N291, N317, N354, and N374	2 Clusterin glycoforms significantly increased after the removal of ccRCC	Gbormittah et al. (2015)
ccRCC	Human plasma	N-Glycopeptide	NA	LC-MS/MS (MRM)	Peak boundary net	39 Differential N-glycopeptides	Differential glyco-isoform abundance of plasma proteins may be a useful source of biomarkers for the clinical course and prognosis of ccRCC	Serie et al. (2022)
RCC	Human serum	N-Glycan	Methyl esterification derivatization and benzyloxyamine labeling	MALDI-MS	Flex analysis	56 N-glycans in RCC patients and HC	Serum N-glycan alteration was associated with RCC	Hatakeyama et al. (2014)
UC	Human serum	N-Glycan	Methyl esterification derivatization and benzyloxyamine labeling	MALDI-MS	GlycoMod	70 N-glycans were identified. 36 N-glycan were quantified with good reproducibility	Serum N-glycan content has the potential to be a specific and sensitive novel diagnostic biomarker in UC	Oikawa et al. (2018)
UC	Human serum Igs	N-Glycan	Methyl esterification derivatization and benzyloxyamine labeling	MALDI-MS	GlycoMod	32 N-glycans were identified in Igs fraction	Aberrant N-glycosylation signatures of Igs were found to be promising diagnostic biomarkers of UC	Tanaka et al. (2017)
ccRCC	Human kidney tissue	N-Glycan	NA	MALDI-MSI	GlycoWorkbench	81 N-glycans in total. 7 N-glycans in the Stage 1 ccRCC. 11 N-glycans in stage 4 ccRCC	Multiple tumor-specific N-glycans were detected with tri- and tetra-antennary structures and varying levels of fucosylation and sialylation	Drake et al. (2020)
Anti-GBM disease	Human MPO	N-Glycopeptide, N-glycoprotein	NA	LC-MS/MS	Byonic	272 N-glycopeptides. 138 N-glycans. 5 N-glycosylation sites: N323, N355, N391, N483, and N729	Atypical glycosylation pattern found on MPO might contribute to its specific processing and presentation as a self-antigen by antigen-presenting cells	Reiding et al. (2019)
ADPKD	α3-Integrin light chain in *Pkd1* ^*+/+*^ and *Pkd1* ^*−/−*^ cells from mouse kidneys	Deglycosylated N-glycopeptide and intact N-glycopeptide	NA	LC-MS/MS	SEQUEST	3 N-glycopeptides. 6 major N-glycans. 4 N-glycosylation sites: N937, N971, N925, and N928	Abnormal protein N-glycosylation (disialylated structures) may have a role in the pathogenesis of cyst formation in ADPKD	Zhang et al. (2014)
PMN	Human sera IgG4	N-Glycopeptide. N-glycan	Sialic acid derivatization	MALDI-MS. LC-MS/MS	LaCyTools	15 N-glycans	An altered glycosylation pattern of IgG4 was identified in PMN patients that facilitated complement activation through the lectin pathway	Haddad et al. (2021)
LN	Human serum IgG	N-Glycans	Permethylation	MALDI-MS	mMass	26 N-glycans	IgG with fucosylation is pathogenic; IgG with galactosylation in LN is nonpathogenic	Bhargava et al. (2021)
LN	Mouse primary mesangial cell lines	Sialic acid-containing N-glycans	NA	MALDI-MS	GlycoWorkbench	19 sialylated N-glycans	NEU may mediate IL-6 release by desialylation glycoproteins	Sundararaj et al. (2021)
LN	Human kidney tissue	N-Glycans	Sialic acid derivatization with dimethylamine	MALDI-MSI	mMass and GlycoWorkbench	58 N-glycans	Increased abundance and spatial distribution of unusual mannose-enriched glycans	Alves et al. (2021)

DKD, diabetic kidney disease; IgA, immunoglobin A; IgAN, IgA nephropathy; AC associated IgAN, alcoholic cirrhosis associated IgAN; RCC, renal cell carcinoma; ccRCC, clear cell RCC; UC, urothelial carcinomas; anti-GBM disease, anti-glomerular basement membrane disease; ADPKD, autosomal dominant polycystic kidney disease; MN, membranous nephropathy; PMN, primary MN; LN, lupus nephritis; HC, healthy control; DM, diabetes mellitus; T2DM, type 2 DM; mIgA, monoclonal IgA; MPO, myeloperoxidase.

### 3.1 N-glycosylation and diabetic kidney disease

Diabetes mellitus (DM) is one of the most common diseases worldwide. Long-term poor glycemic control can lead to various degrees of kidney injury, and 30%–40% of DM patients will progress to DKD (Alicic et al., 2017). Globally, DKD is the leading cause of ESRD and even accounts for 50% of the total ESRD cases in many developed countries (Umanath and Lewis, 2018). According to the clinical course, DKD can be divided into four stages characterized by normoalbuminuria, microalbuminuria, macroalbuminuria, and progressive declining kidney function, respectively. Early intervention is unquestionably beneficial to kidney survival. However, the diagnosis of DKD is usually delayed due to the insidious onset, and the proteinuria will be hard to reverse once it develops into the macroalbuminuria stage. Consequently, it is urgently needed to strengthen the ability of early discovery and aim the key point to improve the outcomes of patients with DKD.

Metabolic disorders in DM and DKD, as the initiating factor of organic lesions, can disturb the normal N-glycosylation by enhancing the hexosamine biosynthesis pathway (Rudman et al., 2019). Different types of DM might lead to differences in the N-glycoproteome. To test this hypothesis, Liljedahl et al. (2016) simulated T1DM and T2DM patients by establishing streptozotocin (STZ)-induced and db/db diabetic mice models. The team profiled the N-glycosylated proteome of the kidney tissue and found that 242, 352 N-glycoproteins were solely in the db/db mouse model and the STZ mouse model, respectively. Among them, some N-glycoproteins expressed with contrary tendency between two models, such as integrin-β1, were increased in the STZ diabetic mice but decreased in the db/db models with significant statistic difference, while the situation of sodium/glucose cotransporter-1 was opposite, which suggested that different etiologies in T1DM and T2DM could lead to variations in the cell adhesion and cell matrix composition. More importantly, differential N-glycoproteins might have higher clinic values than pathological features since they change before the morphological alterations of DKD. As for the study on N-glycoproteomics of DKD patients, unique glycosylation characteristics either in comparison with healthy control (HC) or in each stage of kidney damage have also been uncovered. Zhang et al. (2021) analyzed intact N-glycopeptides of plasma IgG in 28 DKD patients, 48 membranous nephropathy (MN) patients, 35 IgAN patients, and 58 HC by EThcD-sceHCD-MS/MS which could detect more informative fragment ions. With this technical advantage, they reported 72, 73, 83, and 93 unique N-glycopeptides in DKD, MN, IgAN, and HC, respectively. Among them, 28 N-glycopeptides expressing marked differences might be used as potential biomarkers.

Guo et al. (2015) analyzed the total urinary N-glycoproteins between T2DM patients and HC and screened out six differential proteins validated by Western blot, which could be a useful tool to judge the DKD stage and renal function. In this study, alpha-1-antitrypsin and ceruloplasmin were of even greater clinical significance for they could sensitively and specifically differentiate the microalbuminuria stage with significant area under the curve (AUC) values of 0.929 and 1.000, respectively.

**FIGURE 5:**
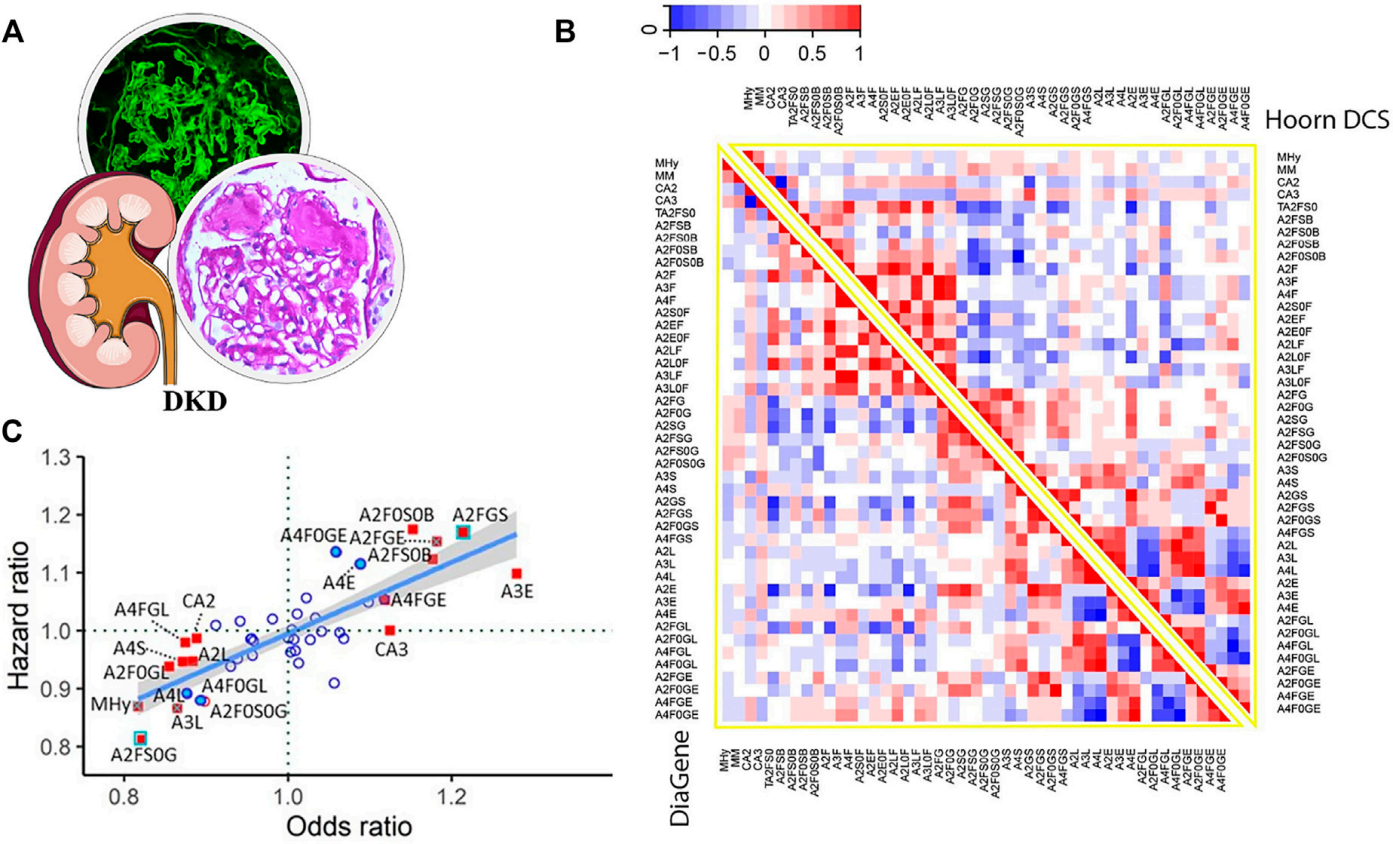
**(A)** Pathological features of DKD. **(B)** Heatmaps displaying the correlations between N-glycan-derived traits in two large T2DM cohorts. **(C)** DKD-associated N-glycan HR plotted versus OR for meta-analyzed data from the two T2DM cohorts (adjusted for age, sex, and age × sex interaction). Red-filled blue square: significant in prevalent and incident complications after FDR correction. Red-filled square with blue cross: significant in prevalent complications after FDR correction and in incident complications before FDR correction. Red-filled square: significant in prevalent complications after FDR correction. Blue-filled circle: significant in incident complications before FDR correction. Red unfilled circle: significant in prevalent complications before FDR correction. Blue unfilled circle: non-significant. Reproduced from Memarian, E., ‘t Hart, L. M. Slieker, R. C., Lemmers, R., van der Heijden, A. A., Rutters, F., et al. (2021). Plasma protein N-glycosylation is associated with cardiovascular disease, nephropathy, and retinopathy in type 2 diabetes. BMJ open diabetes research and care, 9 (1), e002345. doi: 10.1136/bmjdrc-2021-002345 under a Creative Commons CC-BY license (https://creativecommons.org/licenses/by/4.0/).

Poor glycemic control interferes with the process of N-glycosylation; in the meantime, abnormal N-glycosylation can bring alterations in protein functions and in turn contribute to DKD, so N-glycan detection also provides much valuable information on mechanism and diagnosis. Compared to HC, some glycoforms from the plasma of T2DM patients express significant differences and might change the inflammatory state (Dotz et al., 2018). In 2021, Memarian et al. (2021) analyzed total plasma N-glycome patterns in two large independent T2DM cohorts and reported 15 differential N-glycoforms which were associated with DKD after FDR correction (Figure 5). The main N-glycan features, complexity, fucosylation, galactosylation, sialylation, and bisection, were related to DKD closely. Among them, 2,6-sialylation on triantennary glycans presented the most pronounced positive relation with DKD (OR = 1.28, *p* = 9.70 × 10^−6^) and might induce renal damage by promoting inflammation.

### 3.2 N-glycosylation and IgA nephropathy

IgAN is the most prevalent primary glomerular disease in the world. The disease gets its name from the pathological feature, that is, the dominant or codominant mesangial IgA deposition. The incidence of IgAN ranges from 0.2 to 5 per 100,000 individuals per year with geographical differences. In Europe and North America, IgAN is the second leading cause of ESRD only behind DKD, and in East Asian countries, it dominates as the cause of ESRD among various primary glomerulonephritis. In general, nearly 20–40% of IgAN patients will develop ESRD within 10–20 years after diagnosis (Pattrapornpisut et al., 2021). Given the still unknown etiology and the heterogeneous course, it is important to further investigate the pathogenesis and adequately screen the risk biomarkers which might predict poor kidney survival.

**FIGURE 6:**
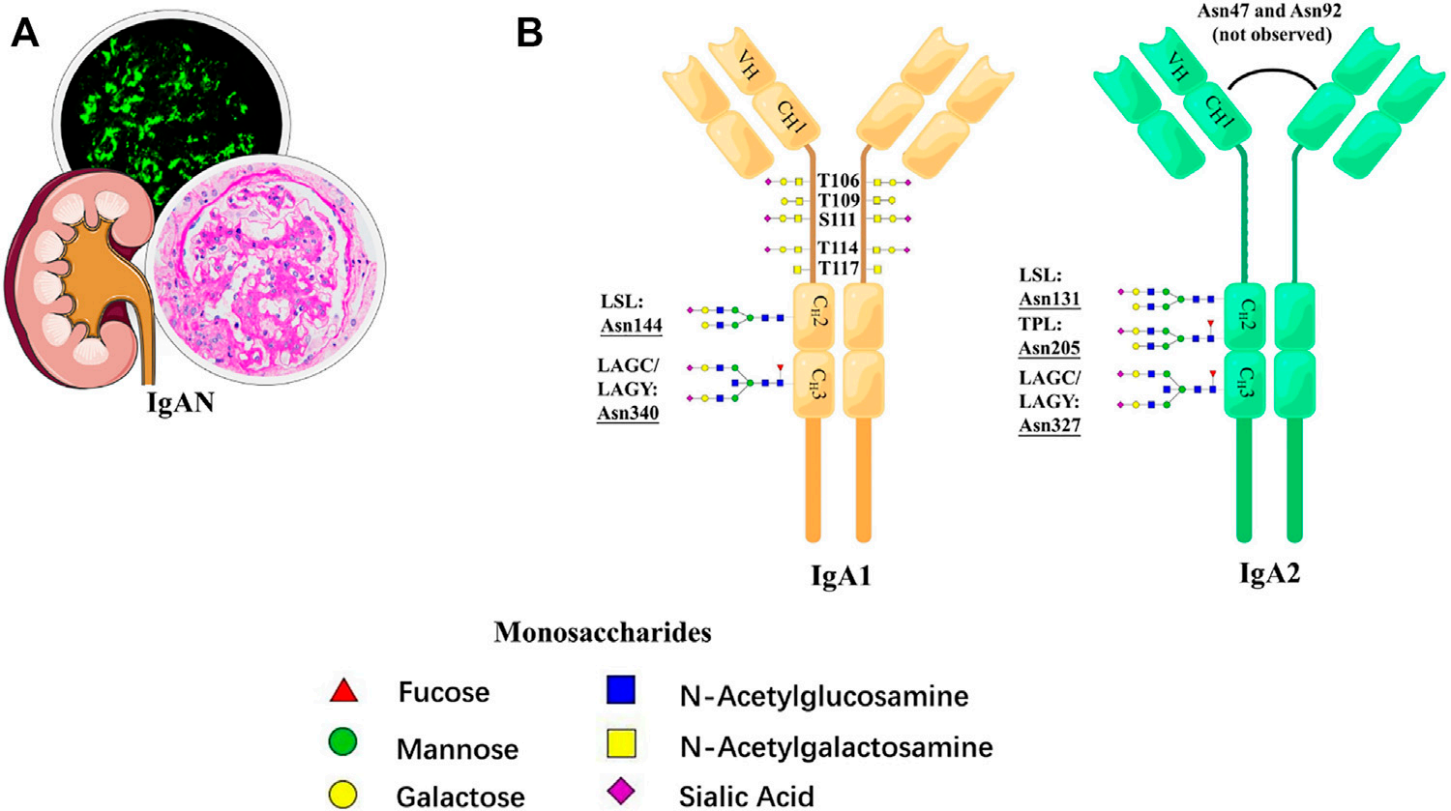
Schematic representation of N-glycosylation in IgAN. **(A)** Pathological features of IgAN. **(B)** N-glycan structures and N-glycosylation sites for IgA1 and IgA2.

Although the formation of the galactose-deficient hinge-region O-glycopeptide is well accepted as the initiating factor in the pathogenesis of IgAN, alteration of N-glycosylation is gradually considered to be involved in the process (Suzuki and Novak, 2021). In 2020, Dotz et al. first elucidated in detail the site-specific N-glycosylation signature of serum IgA1 and IgA2 in IgAN (Figure 6). With the LC-MS approach, they reported, in total, 39 tryptic N-glycopeptides and 36 derived N-glycan traits in 83 patients with IgAN. It is speculated that N-glycosylation participated in the mechanism by upregulating polymeric IgA, which led to the formation and glomerular deposition of the immune complex (IC). Compared with the galactose-deficient O-glycopeptide, the N-glycopeptides and derived traits had better performance in the diagnosis of IgAN because of the higher McFadden-adjusted pseudo-R^2^ value. In addition, the N-glycopeptides and derived traits are associated more closely with deteriorating kidney function, suggesting the possibility of the potential of glycoproteomics in clinical settings (Dotz et al., 2021).

In view of the vital role of N-glycan for the function of IgG Fc domain, abnormal N-glycans in IgA Fc alpha receptor Ⅰ (FcαRⅠ) might be involved in the process of IgAN as well. Göritzer et al. (2019) analyzed FcαRⅠ by LC-MS and found six N-glycosylation sites occupied with different levels. The heterogeneous N-glycans binding to the sites presented site-specific variations in the levels of sialyation, galactosylation, and branching. Furthermore, the FcαRⅠ N-glycans could regulate the binding affinity to IgA significantly, which provided the physiopathological basis for IgAN. A rare IgAN case characterized by monoclonal IgA (mIgA) staining in the mesangium reported by Kaneko et al. (2010) also deepened our understanding of the disease from the perspective of N-glycosylation. Compared with a control patient with circulating mIgA lacking renal involvement and an HC, this patient showed that the mIgA N-glycan profile was abnormal by an in-depth analysis of sera IgA with MALDI-MS. Further study showed that extra N-glycans on the heavy and light chains of IgA disrupted the normal conformation of IgA, which suggested that aberrant N-glycosylation in IgA might be involved in disease progression (Narimatsu et al., 2014). IgA-containing circulating IC, which is the feature of primary IgAN, was also found in the secondary IgAN induced by alcoholic cirrhosis (AC), even in the early stage, suggesting that the two diseases might share some mechanisms in common. To illustrate similarities and differences in N-glycosylation between the two diseases and explore the inner mechanism, research detected the N-glycan profile of IgA1 among patients with compensated or advanced AC, patients with primary IgAN, and HC by MALDI-MS. The result indicated the N-glycans in IgA1 of AC were different from the ones in healthy people and primary IgAN, which might be the result of the decreased level of N-linked carbohydrate clearance by hepatocytes. Aberrant N-glycosylation could modulate the expression of the mesangial transferrin receptor, and thus contributed to the development of AC-associated IgAN directly or indirectly (Novak and Julian, 2011; Tissandié et al., 2011).

### 3.3 N-glycosylation and renal cell carcinoma

RCC, which comprises kinds of pathological types, accounts for 3% of all malignancies in adults. ccRCC representing 80% of the total number of RCCs is the most prevalent one (Escudier et al., 2019). Cancer occurring in the renal pelvis also belongs to urothelial carcinomas (UC) because of the cell type. Although RCC patients in the early stage can be cured by surgery with 5-year survival rates up to 81%, the outcome may be disappointing as the rates would go down to nearly 50% if the tumor is recurrent or the stage is late (Larroquette et al., 2021). Therefore, it is necessary to develop biomarkers for early accurate detection and targets for precise treatment.

Because laboratory tests lack specificity, the diagnosis of RCC is usually established by imaging examinations, such as ultrasound, computed tomography, and magnetic resonance imaging. However, an upgrade of the examination tool is intensively needed to improve prognosis because the visible mass represents probable metastasis and spread. Fortunately, the research on N-glycoproteome in RCC has broadened our vision and may arm clinical workers in the future. In 2020, Santorelli et al. (2020) conducted the first qualitative and quantitative determination of urinary N-glycoproteins in ccRCC patients with different stages and non-ccRCC controls by LC-MS/MS. The result showed that a couple of glycoproteins affecting the occupancy of the glycosylation site differed considerably not only between patients and non-ccRCC controls but also among different cancer stages. Thereinto, three N-glycoproteins were up-expressed in ccRCC patients, including CD97, coagulation factor c homolog, while six N-glycoproteins were down-expressed, such as haptoglobin, fibronectin, and ceruloplasmin, suggesting that urinary N-glycoproteins were related to malignancy and could be applied as noninvasive biomarkers (Ene et al., 2020). Plasma clusterin is heavily glycosylated, and its N-glycan alterations were closely linked to ccRCC before and after curative nephrectomy (Tousi et al., 2012). Gbormittah et al. (2015) investigated site-specific changes of clusterin N-glycans and the sites’ occupancy. In addition to two glycoforms at N374, namely, the biantennary digalactosylated disialylated glycan and the core fucosylated biantennary digalactosylated disialylated glycan, expressed differently before and after curative nephrectomy, they also provided elaborate information of the occupancy and the heterogeneity of each site, which could be used as diagnostic markers and therapeutic targets (Wang et al., 2019). In the meantime, the plasma clusterin along with other four N-glycopeptides was also proved to be strongly associated with the progression-free survival of ccRCC patients treated surgically (hazard ratio range 6.3–11.6, *p* ≤ 0.05), suggesting that differential N-glycopeptides may be predictors for the prognosis of ccRCC as well (Serie et al., 2022).

**FIGURE 7:**
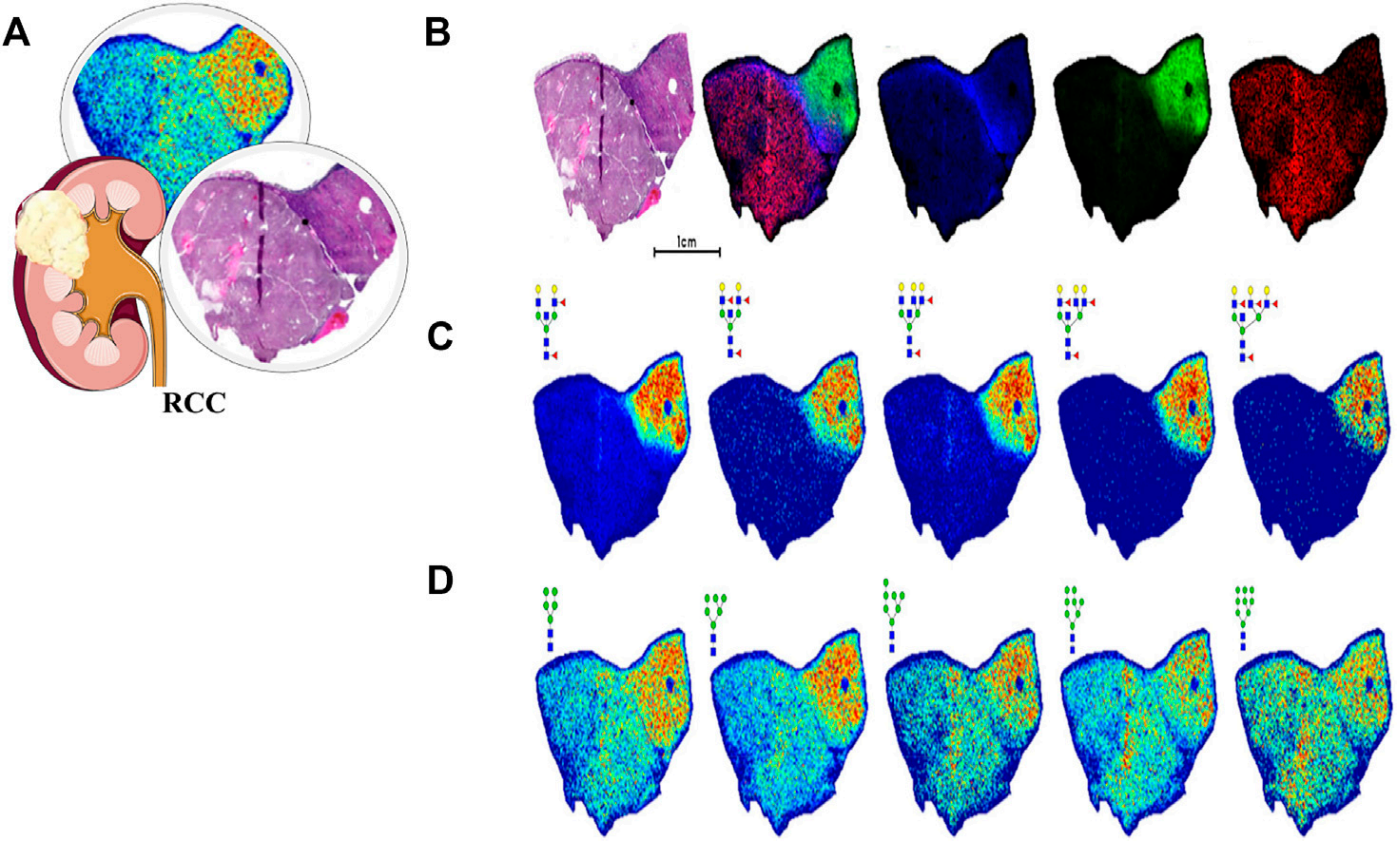
N-glycan imaging MS of a representative ccRCC tissue. Blue for fibrillar, green for nontumor tissue, and red for tumor tissue. **(A)** Pathological features of RCC. **(B)** H and E stain shows with an overlay of three specific regions. **(C)** Multiple N-glycans with bisecting GlcNAc and multiple fucosylated residues localized to the nontumor region. **(D)** Distribution of high-mannose N-glycans. Reproduced from Drake, R. R., McDowell, C., West, C., David, F., Powers, T. W., Nowling, T., et al. (2020). Defining the human kidney N-glycome in normal and cancer tissues using MALDI imaging mass spectrometry. Journal of mass spectrometry: JMS, 55 (4), e4490. doi: 10.1002/jms.4490 under a Creative Commons Attribution 4.0 International License (https://creativecommons.org/licenses/by-nc-nd/4.0/).

Similarly, a view of N-glycomics in malignancy might offer many options in diagnosis and treatment. In 2013, Hatakeyama et al. (2014) first found that the serum N-glycan profile significantly varied between RCC patients and HC, and some of them could get the potential to predict overall survival. Then in 2016, with the help of MALDI-MS, the same team further singled out six N-glycans from serum proteins and established a diagnostic score for UC with a sensitivity, specificity, and AUC of 93%, 81%, and 0.95, respectively, which was more sensitive than the traditional method of urine cytology (Oikawa et al., 2018). Serum immunoglobulins are heavily glycosylated and provide the majority of aberrant N-glycans. The team continued analyzing the N-glycomics of Igs in UC patients’ serum, and then established a diagnostic score combining five out of 32 types of UC-associated N-glycans with a sensitivity, specificity, and AUC of 92.8%, 97.2%, and 0.969, respectively, which was far more superior to classic urine cytology (AUC, 0.707) and even could be applied in patients without hematuria (Tanaka et al., 2017). In addition, a large-scale, multi-center quantitative study of serum IgG glycosylation in 12 organs’ cancer cohorts including the kidney showed that galactosylation was distributed with a statistically significant difference. Moreover, a Gal-ratio of IgG comprising three galactosylations was established, which could distinguish cancer cases from non-cancer controls noninvasively (AUC, >0.8), even in their early stage (Ren et al., 2016). Using an imaging MS approach, different characteristics between the ccRCC region and normal kidney tissue can be assessed. In the ccRCC regions, multi-antennary structures with different levels of sialylation and fucosylation were detected, while in the normal regions, such as the proximal tubule, N-glycans with bisecting GlcNAc and a high level of fucosylation were dominant. N-glycans from the representative tissue of ccRCC visualized using MALDI-MSI is shown in Figure 7. This figure shows that glycan distributions differ among tumor regions and normal regions, which can supplement the pathological diagnosis. The research promoted further research on the role of tissue N-glycome in renal cancer and paved the way for glycobiology in the clinical field (Drake et al., 2020).

### 3.4 N-glycosylation and other kidney diseases

Compelling evidence based on MS has clearly demonstrated that abnormal N-glycosylation is closely related to autoantigen formation, protein conformational change, complement activation, and signaling pathway interference. Therefore, in addition to the aforementioned common benign and malignant diseases, N-glycosylation is also of great significance to some autoimmune and genetic kidney diseases, such as anti-GBM disease, ADPKD, PMN, and LN.

**FIGURE 8:**
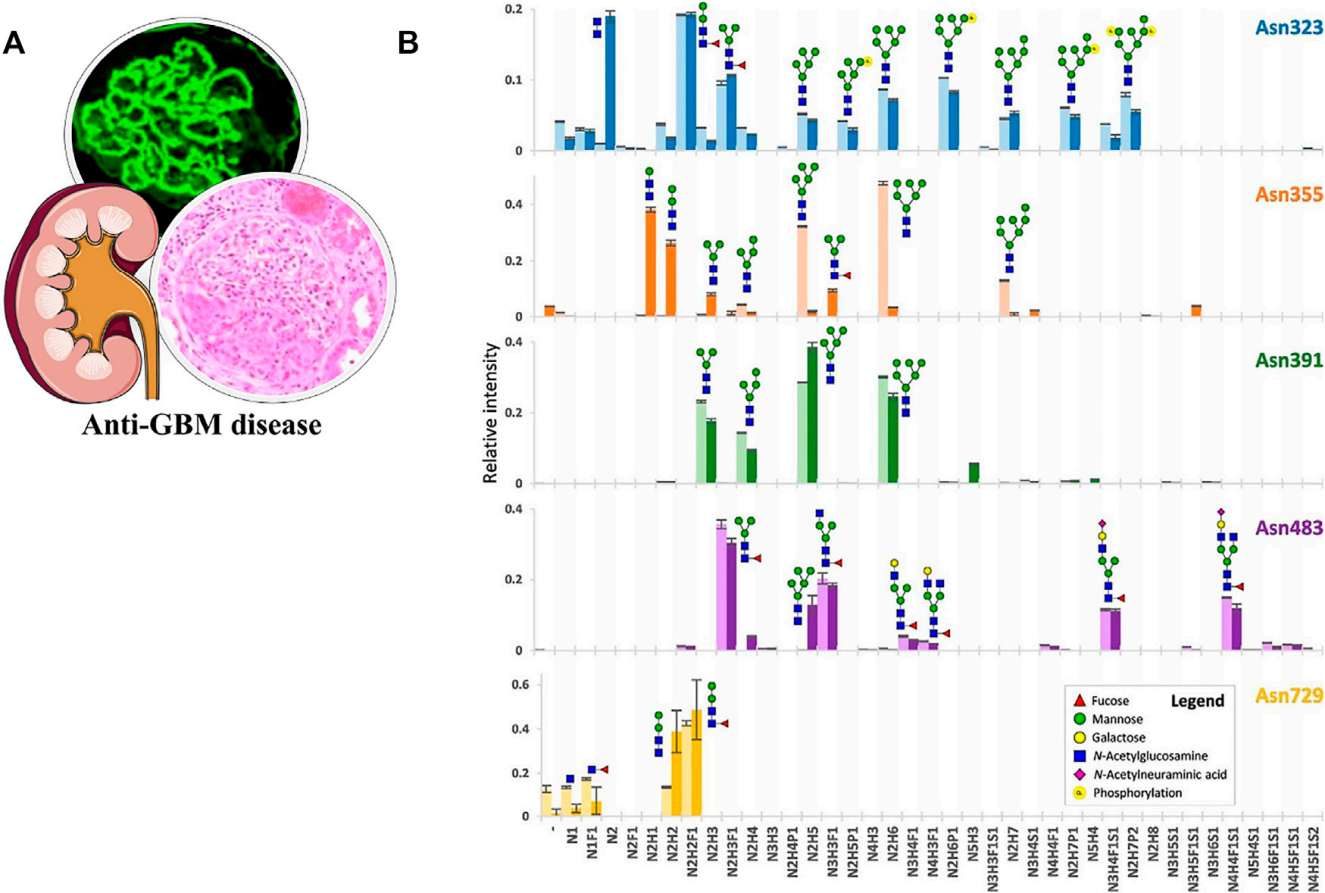
**(A)** Pathological features of the anti-GBM disease. **(B)** Qualitative and quantitative overview of glycan distribution per MPO N-glycosylation site. Each colored bar represents one N-glycosylation site, the lighter and darker variant, respectively, the biologically independent discovery pool and replication pool. The height of the bars indicates the mean relative area of MS^1^ signals from a triplicate LC-MS^2^ run (the MS^2^ differing in the fragmentation scheme) and the error bars the S.D. thereof. Reproduced from Reiding K. R, Franc V, Huitema M. G, Brouwer E, Heeringa P, Heck A. J. R. (2019). Neutrophil myeloperoxidase harbors distinct site-specific peculiarities in its glycosylation. J Biol Chem. 294 (52):20,233–20,245. doi: 10.1074/jbc.RA119.011098 under a Creative Commons CC-BY license (https://creativecommons.org/licenses/by/4.0/).

Anti-GBM disease is an IC-mediated vasculitis featured by the presence of anti-GBM autoantibodies and rapidly declining kidney function. The common view has long held that only 20–30% of patients with the anti-GBM disease have the detectable antineutrophil cytoplasmic antibody, especially against myeloperoxidase (MPO), namely, the “double positive” (Hellmark et al., 1997). However, Li et al. (2016) proved that although the positive rate of antibodies against intact MPO in anti-GBM cases was indeed less than 30%, up to 60% recognized linear epitopes of MPO. Moreover, some of the antibodies were associated with disease severity, suggesting the significance of these linear epitopes. Reiding et al. (2019) first uncovered the heterogeneity of N-glycosylation in MPO and the possibility of the glycan epitopes. They performed a qualitative and quantitative analysis of N-glycans in MPO with bottom-up glycoproteomics and native MS approaches. The remarkably high diversity of glycans in the five sites and the occupancy levels were unveiled (Figure 8). In addition, they also reported highly truncated paucimannose species in the extracellular space. Yu et al. (2017) first directly proved that aberrant N-glycosylation could lead to the exposure of neo-epitopes in MPO. They prepared six atypical glycosylated MPO molecules with all possible glycosylation types and found that these molecules could bind to sera antibodies from 21 patients with the anti-GBM disease (40 patients in total). Among different types, the antigens with five peptides containing one site of N323, N355, or N391 presented high specificity to 90% of these antibodies. These findings of N-glycosylation in MPO deepened the understanding of anti-GBM disease undoubtedly.

ADPKD is the most frequent monogenetic disease in humans (Cornec-Le Gall et al., 2019). Variants in *PKD1* and *PKD2* which account for the major cause of ADPKD are strongly associated with abnormal N-glycosylation. Zhang et al. (2014) conducted a detailed analysis of N-glycosylation of α3 integrin in *Pkd1* ^*+/+*^ and *Pkd1* ^*−/−*^ cells from mouse kidneys using the glycoproteomics approach. The glycopeptides in Asn-925 and Asn-928 sites of *Pkd1* ^*+/+*^ have higher molecular weight, and unique disialic acid glycan structures were only observed in *Pkd1* ^*−/−*^ cells, suggesting that abnormal N-glycosylation might take part in cyst formation. Polycystin-2 (PC-2) encoded by *PKD2* is a calcium-activated cation transient receptor potential channel. Data from a cryo-electron microscope indicated two distinct structures of PC-2 which reveal conformational differences in the selectivity filter and in the large exoplasmic domain. Differing N-glycosylation sites and the motifs they formed in the homomeric PC-2 complex might trigger the conformational changes, influence the function of the selectivity filter, and further provide information on the structural effects of ADPKD mutations (Wilkes et al., 2017).

PMN is an organ-specific autoimmune kidney disease caused by circulating autoantibodies mainly against M-type phospholipase A2 receptor (PLA2R) on glomerular podocytes (Hoxha et al., 2022). Haddad et al. (2021) reproduced the complement activation and podocyte injury in cultured cells with sera IgG4 from PMN patients and found that the complement was activated in a N-glycosylation-dependent manner, while deglycosylation would lose the pathogenicity of autoantibody IgG4. A further profile of the glycoforms of pathogenic IgG4 suggested a significant increase in the galactose-deficient patterns with a corresponding decrease in galactosylated ones compared with HC by LC-MS. Moreover, these aberrant IgG4 glycoforms also correlated with the anti-PLA2R antibody levels and with the degradation of vital podocyte structural proteins. Therefore, these findings shed light on the N-glycosylation of autobody in the pathogenesis of PMN.

N-glycosylation participates in the pathogenetic process of LN by various mechanisms. Compared with HC, calcium/calmodulin kinase IV (CaMK4), which can target podocytes by restraining nephrin transcription, expresses increasingly in podocytes of LN patients (Maeda et al., 2018). With the method of MALDI-MS, Bhargava et al. (2021) first described the critical role of N-glycans that related to CaMK4 closely. Specifically, fucose on N-glycans of IgG from LN patients could increase the level of CaMK4, whereas galactosylation had the opposite effect. Neuraminidase (NEU) can induce glomerular inflammation and promote tissue damage in LN by regulating the secretion of IL-6 (Sundararaj et al., 2018). A further study uncovered that NEU could decrease the level of sialic acid-containing N-glycans to unmask the receptors by which TLR4 activated TLR4-p38/ERK MAPK signaling in primary mouse mesangial cells (Sundararaj et al., 2021). Aberrant tissue glycans also contributed to immunogenicity in LN. Alves et al. (2021) first reported the abnormally increased and distributed glycosylation pattern of cellular mannose-enriched glycans in the kidneys of LN patients by MALDI-MSI. These abnormal mannosylated-glycans caused by the downregulation of complex N-glycosylation pathways could act as glycan-epitopes, be recognized by specific glycan-recognizing receptors of immune cells, and then trigger the immune response.

## 4 Outlook and future directions

Kidney disease is not only an increasingly huge burden for global health but also the most complex condition which needs more attention and research efforts (Tonelli et al., 2018). The rapid development of MS enables us a deeper understanding of kidney disease from the view of glycoproteomics and glycomics. As discussed previously, changes in N-glycosylation in some types of kidney diseases would create new opportunities to expand and diversify the repertoire of biomarkers in early diagnosis, course observation, and prognosis evaluation. In the meantime, several exciting achievements have unveiled the value of therapeutics targeting N-glycosylation *in vitro* and animal-based studies. For example, inhibiting core fucosylation could protect the peritoneal mesothelial cell from the epithelial–mesenchymal transition (Li et al., 2017; Li et al., 2018; Yu et al., 2021). It might delay the gradual decline of dialysis adequacy caused by peritoneal fibrosis in long-term peritoneal dialysis. Likewise, glycoengineering which can restructure the N-glycans in Klotho might help achieve better optimal receptor activity and quality control for the anti-aging membrane protein (Zhong et al., 2020). These findings provide a new train of thought and tactics for treating kidney disease undoubtedly.

Regarding global N-glycoproteome/N-glycome or N-glycoproteins of interest, site-specific N-glycan analysis that could provide relatively comprehensive information on protein N-glycosylation is attracting much attention. However, there are still some issues to be addressed, such as linkage type, fucose migration, and the interpretation of mixed glycopeptide spectra. The characterization of clinically relevant glycoproteoform at intact protein levels, which was able to offer faster and reproducible workflow, is also a promising approach.

Regarding the development of MS-based workflow, it is essential to develop highly specific enrichment methods, highly efficient fragmentation, high speed, and highly precise glyco-informatics for in-depth N-glycoproteome profiling or detailed characterization of key N-glycoproteins in large cohort samples of kidney disease. IM-MS and CE-MS, as powerful separation techniques were able to resolve isomeric glycans, but they have not been deeply explored for N-glycosylation analysis in kidney disease. The emerging non-targeted MS acquisition mode, data-independent acquisition (DIA), was still in its infancy for N-glycoproteomics. More recently, GproDIA enabled the accurate identification of intact glycopeptides, demonstrating great potential in high-throughput clinical N-glycosylation analysis (Yang et al., 2021). The establishment of an integrated high-throughput clinical MS platform would be of great importance.

Although MS-based N-glycosylation analysis was focused on in this review, aberrant O-glycosylation was also found to be closely associated with kidney disease. Recently, an MS-based method called extraction of O-glycopeptides (EXoO) was developed, which enabled large-scale identification of O-glycosylation sites and site-specific O-glycans in mucin-type glycoproteins. By EXoO, expression differences in the O-glycoproteome of normal and tumor kidney tissues were revealed (Yang et al., 2018). Similar to MS-based N-glycosylation analysis, improvements in enrichment and separation strategies, MS dissociation methods, and software tools will be valuable for in-depth O-glycosylation analysis in high-throughput, thereby providing more comprehensive data for the diagnosis and treatment of kidney disease.

Overall, we believe that the dramatic improvements in N-glycosylation analytical technologies and methods will contribute greatly to a better understanding of the mechanisms through which kidney disease progresses, the discovery of more sensitive diagnosis and prognosis candidate biomarkers, and the innovation of more effective therapeutic targets.

## Glossary

2-AA: 2-aminobenzoic acid
AC: alcoholic cirrhosis
ADPKD: autosomal dominant polycystic kidney disease
AI-ETD: activated-ion electron transfer dissociation
Asn: asparagine
AUC: area under the curve
CaMK4: calcium/calmodulin kinase IV
ccRCC: clear cell renal cell carcinoma
CKD: chronic kidney disease
DIA: data-independent acquisition
DKD: diabetic kidney disease
DM: diabetes mellitus
DMEN: N,N-dimethylethylenediamine
ESRD: end-stage renal disease
ETD: electron transfer dissociation
EThcD: electron-transfer/higher-energy collisional dissociation
EXoO: extraction of O-glycopeptides
FcαRⅠ: Fc alpha receptor Ⅰ
FDR: false discovery rate
GBM: glomerular basement membrane
GlcNAc: N-acetylglucosamine
HC: healthy control
HCD: higher-energy collisional dissociation
HILIC: hydrophilic interaction chromatography
IC: immune complex
IM-MS: ion mobility MS
IgA: immunoglobin A
IgAN: IgA nephropathy
HPLC: high-performance liquid chromatography
LN: lupus nephritis
mIgA: monoclonal IgA
MN: membranous nephropathy
MPO: myeloperoxidase
MS: mass spectrometry
MSI: MS imaging
NEU: neuraminidase
PC-2: polycystin-2
PLA2R: M-type phospholipase A2 receptor
PMN: primary membranous nephropathy
PNGase: peptide-N-glycosidase
RCC: renal cell carcinoma
sceHCD: stepped collision energy HCD
STZ: streptozotocin
Ser: serine
T1DM: type 1 diabetes mellitus
T2DM: type 2 diabetes mellitus
Thr: threonine
UC: urothelial carcinomas
